# *N*-[(2-Chloro­phen­yl)sulfon­yl]-3-nitro­benzamide

**DOI:** 10.1107/S1600536813015912

**Published:** 2013-06-12

**Authors:** S. Sreenivasa, D Darshan, T. N. Lohith, G. R. Mamatha, B. S. Palakshamurthy, P. A. Suchetan

**Affiliations:** a Department of Studies and Research in Chemistry, Tumkur University, Tumkur, Karnataka 572 103, India; b University College of Science, Tumkur University, Tumkur, India; c Department of Studies and Research in Physics, U.C.S., Tumkur University, Tumkur, Karnataka 572 103, India; d Department of Chemistry, University College of Science, Tumkur University, Tumkur 572 103, India

## Abstract

In the title compound, C_13_H_9_ClN_2_O_5_S, the dihedral angle between the benzene rings is 74.86 (11)°. The mol­ecule is twisted at the S atom, with a dihedral angle of 82.53 (13)° between the sulfonyl benzene ring and the S—N—C=O segment. In the crystal, mol­ecules are linked into inversion dimers through pairs of N—H⋯O hydrogen bonds, thereby forming *R*_2_^2^(8) loops. Mol­ecules are linked into *C*(7) [010] chains by weak C—H⋯O hydrogen bonds, and C—H⋯π inter­actions are also observed.

## Related literature

For similar structures, see: Gowda *et al.* (2009 ▶, 2010 ▶); Suchetan *et al.* (2011 ▶, 2012 ▶).
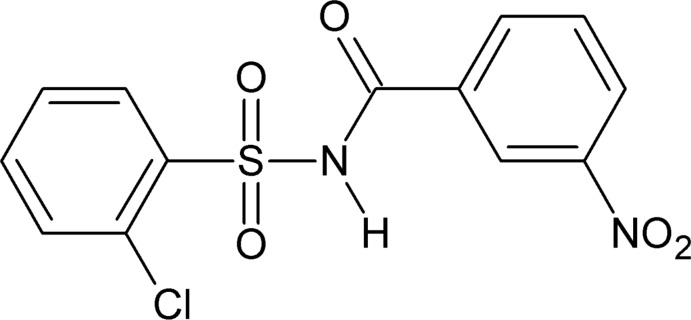


## Experimental

### 

#### Crystal data

C_13_H_9_ClN_2_O_5_S
*M* *_r_* = 340.73
Orthorhombic, 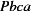

*a* = 12.290 (8) Å
*b* = 13.548 (9) Å
*c* = 17.240 (11) Å
*V* = 2870 (3) Å^3^
*Z* = 8
Mo *K*α radiation
μ = 0.44 mm^−1^
*T* = 298 K
0.32 × 0.26 × 0.18 mm

#### Data collection

Bruker APEXII diffractometer
22957 measured reflections
2523 independent reflections
2170 reflections with *I* > 2σ(*I*)
*R*_int_ = 0.059

#### Refinement

*R*[*F* ^2^ > 2σ(*F* ^2^)] = 0.039
*wR*(*F* ^2^) = 0.095
*S* = 1.06
2523 reflections
203 parameters
H atoms treated by a mixture of independent and constrained refinement
Δρ_max_ = 0.19 e Å^−3^
Δρ_min_ = −0.34 e Å^−3^

### 

Data collection: *APEX2* (Bruker, 2009 ▶); cell refinement: *APEX2* and *SAINT-Plus* (Bruker, 2009 ▶); data reduction: *SAINT-Plus* and *XPREP* (Bruker, 2009 ▶); program(s) used to solve structure: *SHELXS97* (Sheldrick, 2008 ▶); program(s) used to refine structure: *SHELXL97* (Sheldrick, 2008 ▶); molecular graphics: *Mercury* (Macrae *et al.*, 2008 ▶); software used to prepare material for publication: *SHELXL97*.

## Supplementary Material

Crystal structure: contains datablock(s) I, global. DOI: 10.1107/S1600536813015912/hb7091sup1.cif

Structure factors: contains datablock(s) I. DOI: 10.1107/S1600536813015912/hb7091Isup2.hkl

Supplementary material file. DOI: 10.1107/S1600536813015912/hb7091Isup3.cml

Additional supplementary materials:  crystallographic information; 3D view; checkCIF report

## Figures and Tables

**Table 1:** Hydrogen-bond geometry (Å, °) *Cg* is the centroid of the nitro­benzene ring.

*D*—H⋯*A*	*D*—H	H⋯*A*	*D*⋯*A*	*D*—H⋯*A*
N1—H1*N*1⋯O2^i^	0.81 (2)	2.15 (2)	2.954 (3)	169 (2)
C11—H11⋯O3^ii^	0.93	2.50	3.411 (3)	166
C6—H6⋯*Cg* ^iii^	0.93	2.72	3.550 (3)	150

Symmetry codes: (i) 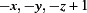; (ii) 
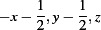
; (iii) 
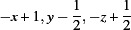
.

**Fig. 1.**
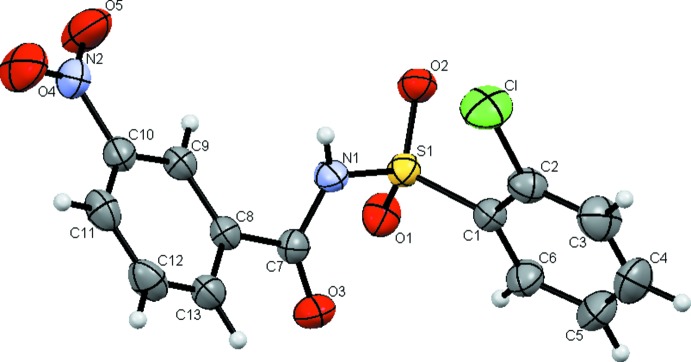
Molecular structure of the title compound, showing displacement ellipsoids drawn at the 50% probability level.

**Fig. 2.**
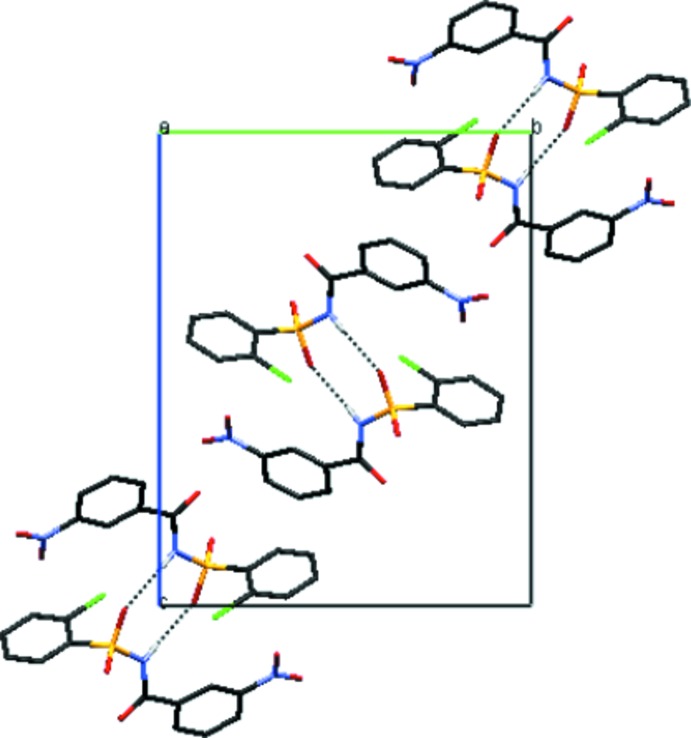
Molecular packing of (I) with hydrogen bonding shown as dashed lines. Carbon bounded hydrogen atoms are ommitted for clarity.

**Fig. 3.**
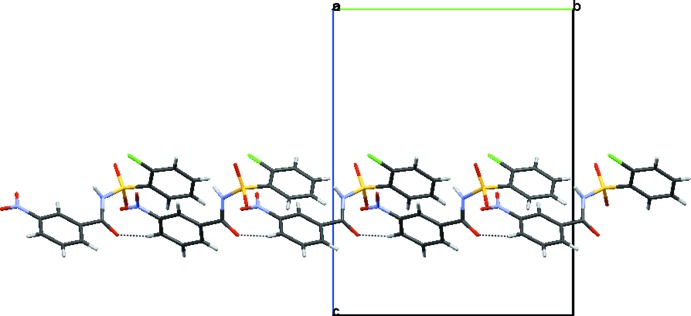
Display of C—H···O interactions among molecules along b axis forming C(7) chains.

**Fig. 4.**
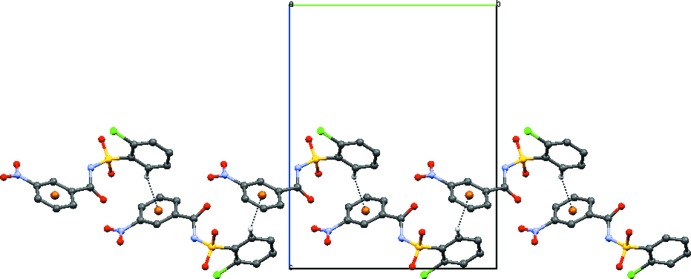
Stacking of molecules along b axis through C—H···Cg interactions. Cg is the centroid of the nitrobenzene ring

## supplementary crystallographic information

### Comment

Diaryl acylsulfonamides are known as potent anti-tumor agents against a broad spectrum of human tumor xenografts (colon, lung, breast, ovary, and prostate) in nude mice. As part of our studies in this area, the structure of the title compound, (I), was determined.

In the title compound, C_13_H_9_ClN_2_O_5_S (I), the conformation of the N—H bond in the C—SO_2_—NH—C(O) segment is anti to the C=O bond. In the molecule, the conformation between the N—H bond and the *ortho*-chloro group in the sulfonyl benzene ring is *syn*. Similarly, the conformation between the N—H bond and the *meta*-nitro group in the benzoyl ring is *syn*. The dihedral angle between the two benzene rings is 74.86 (11)°, compared to the value of 80.3 (1)° in *N*-(benzoyl)-benzenesulfonamide (Gowda *et al.* 2009), 86.9 (2)*^o^ N*-(3-nitrobenzoyl)-benzenesulfonamide (Suchetan *et al.* 2012), 73.3 (1)° in *N*-benzoyl-2-chlorobenzenesulfonamide (Gowda *et al.* 2010) and 85.4 (1)*^o^* in 2-chloro-*N*-(4-nitrobenzoyl)benzenesulfonamide (Suchetan *et al.* 2011). In the crystal, the molecules are linked into inversion dimers through N—H···O hydrogen bonds forming a *R*_2_^2^(8) motif. The molecules are further linked along [010] through a weak C—H···O intermolecular interaction into C(7) chains. C—H···*Cg* (where *Cg* is the centroid of the nitrobenzene ring) interactions are also observed in the crystal structure.

### Experimental

The title compound was prepared by refluxing a mixture of 3-nitrobenzoic acid, 2-chlorobenzene sulfonamide and phosphorous oxy chloride for 2 h on a water bath. The resultant mixture was cooled and poured into ice cold water. The Solid obtained was filtered and washed thoroughly with water and then dissolved in sodium bicarbonate solution. The compound was later reprecipitated by acidifying the filtered solution with dilute HCl. The filtered and dried solid was recrystallized to the constant melting point (483 K).

Colorless prisms of (I) were obtained from a slow evaporation of its ethanolic solution at room temperature.

### Refinement

The H atom of the NH group was located in a difference map and later restained to N—H = 0.81 (2) Å. The other H atoms were positioned with idealized geometry using a riding model with C—H = 0.93 Å. All H atoms were refined with isotropic displacement parameters (set to 1.2 times of the *U*_eq_ of the parent atom).

### Crystal data

**Table:**

C_13_H_9_ClN_2_O_5_S	Prism
*M**_r_* = 340.73	*D*_x_ = 1.577 Mg m^−^^3^
Orthorhombic, *P**b**c**a*	Melting point: 483 K
Hall symbol: -P 2ac 2ab	Mo *K*α radiation, λ = 0.71073 Å
*a* = 12.290 (8) Å	Cell parameters from 456 reflections
*b* = 13.548 (9) Å	θ = 2.4–25.0°
*c* = 17.240 (11) Å	µ = 0.44 mm^−^^1^
*V* = 2870 (3) Å^3^	*T* = 298 K
*Z* = 8	Prism, colourless
*F*(000) = 1392	0.32 × 0.26 × 0.18 mm

### Data collection

**Table:**

Bruker APEXII diffractometer	2170 reflections with *I* > 2σ(*I*)
Radiation source: fine-focus sealed tube	*R*_int_ = 0.059
Graphite monochromator	θ_max_ = 25.0°, θ_min_ = 2.4°
phi and ω scans	*h* = −14→14
22957 measured reflections	*k* = −16→16
2523 independent reflections	*l* = −20→20

### Refinement

**Table:**

Refinement on *F*^2^	Primary atom site location: structure-invariant direct methods
Least-squares matrix: full	Secondary atom site location: difference Fourier map
*R*[*F*^2^ > 2σ(*F*^2^)] = 0.039	Hydrogen site location: inferred from neighbouring sites
*wR*(*F*^2^) = 0.095	H atoms treated by a mixture of independent and constrained refinement
*S* = 1.06	*w* = 1/[σ^2^(*F*_o_^2^) + (0.0417*P*)^2^ + 1.3036*P*] where *P* = (*F*_o_^2^ + 2*F*_c_^2^)/3
2523 reflections	(Δ/σ)_max_ = 0.009
203 parameters	Δρ_max_ = 0.19 e Å^−^^3^
0 restraints	Δρ_min_ = −0.34 e Å^−^^3^

### Special details

**Table:**

Geometry. All s.u.'s (except the s.u. in the dihedral angle between two l.s. planes) are estimated using the full covariance matrix. The cell s.u.'s are taken into account individually in the estimation of s.u.'s in distances, angles and torsion angles; correlations between s.u.'s in cell parameters are only used when they are defined by crystal symmetry. An approximate (isotropic) treatment of cell s.u.'s is used for estimating s.u.'s involving l.s. planes.
Refinement. Refinement of *F*^2^ against ALL reflections. The weighted *R*-factor *wR* and goodness of fit *S* are based on *F*^2^, conventional *R*-factors *R* are based on *F*, with *F* set to zero for negative *F*^2^. The threshold expression of *F*^2^ > 2σ(*F*^2^) is used only for calculating *R*-factors(gt) *etc*. and is not relevant to the choice of reflections for refinement. *R*-factors based on *F*^2^ are statistically about twice as large as those based on *F*, and *R*- factors based on ALL data will be even larger.

### Fractional atomic coordinates and isotropic or equivalent isotropic displacement parameters (Å^2^)

**Table:**

	*x*	*y*	*z*	*U*_iso_*/*U*_eq_	
H1N1	−0.0482 (19)	0.0080 (17)	0.5829 (13)	0.038 (7)*	
Cl	−0.15389 (6)	0.15122 (5)	0.47697 (4)	0.0619 (2)	
O1	0.14765 (12)	0.14055 (11)	0.63851 (9)	0.0459 (4)	
O2	0.08993 (13)	0.09375 (11)	0.50819 (9)	0.0417 (4)	
O3	−0.04676 (15)	0.10265 (12)	0.73735 (9)	0.0500 (4)	
C7	−0.06345 (17)	0.03864 (15)	0.69067 (12)	0.0360 (5)	
C1	−0.01089 (17)	0.23801 (15)	0.57672 (11)	0.0326 (5)	
N1	−0.02055 (16)	0.04195 (13)	0.61651 (11)	0.0372 (5)	
C9	−0.10982 (18)	−0.13960 (15)	0.66930 (12)	0.0354 (5)	
H9	−0.0518	−0.1458	0.6350	0.042*	
C2	−0.09947 (18)	0.24942 (17)	0.52799 (12)	0.0406 (5)	
C8	−0.13061 (17)	−0.05097 (15)	0.70592 (11)	0.0333 (5)	
O4	−0.21793 (19)	−0.37931 (13)	0.65266 (13)	0.0796 (7)	
N2	−0.15398 (19)	−0.31165 (14)	0.64382 (12)	0.0524 (6)	
C3	−0.1469 (2)	0.3409 (2)	0.51955 (15)	0.0565 (7)	
H3	−0.2065	0.3487	0.4868	0.068*	
C12	−0.2825 (2)	−0.12449 (18)	0.77063 (14)	0.0471 (6)	
H12	−0.3407	−0.1189	0.8048	0.057*	
C13	−0.21677 (19)	−0.04398 (17)	0.75771 (12)	0.0427 (6)	
H13	−0.2300	0.0150	0.7836	0.051*	
C10	−0.17671 (19)	−0.21855 (15)	0.68463 (12)	0.0378 (5)	
C5	−0.0202 (2)	0.40986 (18)	0.60766 (16)	0.0587 (7)	
H5	0.0062	0.4642	0.6347	0.070*	
C6	0.0285 (2)	0.31882 (16)	0.61681 (14)	0.0452 (6)	
H6	0.0878	0.3119	0.6499	0.054*	
O5	−0.0744 (2)	−0.31613 (14)	0.60277 (13)	0.0822 (7)	
C11	−0.2639 (2)	−0.21325 (18)	0.73390 (13)	0.0443 (6)	
H11	−0.3089	−0.2674	0.7423	0.053*	
C4	−0.1068 (2)	0.4203 (2)	0.55921 (16)	0.0629 (8)	
H4	−0.1389	0.4820	0.5530	0.076*	
S1	0.06284 (4)	0.12695 (4)	0.58416 (3)	0.03356 (17)	

### Atomic displacement parameters (Å^2^)

**Table:**

	*U*^11^	*U*^22^	*U*^33^	*U*^12^	*U*^13^	*U*^23^
Cl	0.0569 (4)	0.0694 (5)	0.0596 (4)	−0.0047 (3)	−0.0229 (3)	−0.0149 (3)
O1	0.0407 (9)	0.0430 (9)	0.0540 (10)	−0.0002 (7)	−0.0146 (8)	0.0011 (7)
O2	0.0465 (9)	0.0391 (8)	0.0394 (9)	−0.0032 (7)	0.0112 (7)	−0.0044 (7)
O3	0.0670 (12)	0.0416 (9)	0.0415 (9)	−0.0036 (8)	0.0007 (8)	−0.0112 (8)
C7	0.0422 (12)	0.0328 (11)	0.0328 (12)	0.0053 (9)	−0.0023 (10)	−0.0001 (9)
C1	0.0361 (11)	0.0314 (11)	0.0301 (11)	−0.0002 (9)	−0.0001 (9)	0.0002 (9)
N1	0.0493 (12)	0.0319 (10)	0.0305 (10)	−0.0089 (9)	0.0011 (9)	−0.0029 (8)
C9	0.0402 (12)	0.0358 (12)	0.0301 (11)	0.0001 (9)	0.0034 (10)	0.0031 (9)
C2	0.0395 (12)	0.0466 (13)	0.0356 (12)	0.0014 (10)	−0.0034 (10)	−0.0027 (10)
C8	0.0407 (12)	0.0326 (11)	0.0267 (11)	0.0025 (9)	−0.0007 (9)	0.0039 (8)
O4	0.1089 (17)	0.0447 (11)	0.0853 (15)	−0.0325 (11)	0.0239 (13)	−0.0087 (10)
N2	0.0765 (16)	0.0340 (11)	0.0467 (12)	−0.0083 (11)	0.0071 (12)	0.0016 (9)
C3	0.0521 (16)	0.0654 (18)	0.0521 (16)	0.0171 (13)	−0.0112 (13)	0.0027 (13)
C12	0.0413 (13)	0.0607 (16)	0.0392 (13)	0.0037 (12)	0.0109 (11)	0.0101 (11)
C13	0.0540 (14)	0.0412 (12)	0.0331 (12)	0.0089 (11)	0.0054 (11)	0.0042 (10)
C10	0.0467 (13)	0.0345 (11)	0.0321 (11)	−0.0027 (10)	−0.0023 (10)	0.0030 (9)
C5	0.084 (2)	0.0343 (13)	0.0581 (17)	0.0027 (13)	−0.0037 (15)	−0.0073 (12)
C6	0.0565 (15)	0.0357 (12)	0.0433 (14)	−0.0024 (11)	−0.0087 (11)	−0.0038 (10)
O5	0.1074 (18)	0.0453 (11)	0.0940 (16)	−0.0102 (11)	0.0529 (14)	−0.0155 (10)
C11	0.0459 (14)	0.0475 (14)	0.0395 (12)	−0.0067 (11)	0.0023 (11)	0.0109 (11)
C4	0.079 (2)	0.0454 (15)	0.0646 (18)	0.0224 (14)	0.0006 (16)	0.0020 (13)
S1	0.0352 (3)	0.0302 (3)	0.0353 (3)	−0.0017 (2)	−0.0007 (2)	−0.0008 (2)

### Geometric parameters (Å, º)

**Table:**

Cl—C2	1.729 (2)	O4—N2	1.217 (3)
O1—S1	1.4137 (17)	N2—O5	1.209 (3)
O2—S1	1.4243 (17)	N2—C10	1.471 (3)
O3—C7	1.201 (3)	C3—C4	1.367 (4)
C7—N1	1.384 (3)	C3—H3	0.9300
C7—C8	1.491 (3)	C12—C13	1.376 (3)
C1—C6	1.382 (3)	C12—C11	1.378 (3)
C1—C2	1.384 (3)	C12—H12	0.9300
C1—S1	1.761 (2)	C13—H13	0.9300
N1—S1	1.639 (2)	C10—C11	1.370 (3)
N1—H1N1	0.81 (2)	C5—C4	1.360 (4)
C9—C10	1.375 (3)	C5—C6	1.380 (3)
C9—C8	1.381 (3)	C5—H5	0.9300
C9—H9	0.9300	C6—H6	0.9300
C2—C3	1.378 (3)	C11—H11	0.9300
C8—C13	1.388 (3)	C4—H4	0.9300
			
O3—C7—N1	122.1 (2)	C11—C12—H12	119.3
O3—C7—C8	124.3 (2)	C12—C13—C8	119.9 (2)
N1—C7—C8	113.60 (18)	C12—C13—H13	120.1
C6—C1—C2	119.4 (2)	C8—C13—H13	120.1
C6—C1—S1	117.40 (17)	C11—C10—C9	123.1 (2)
C2—C1—S1	122.98 (16)	C11—C10—N2	119.4 (2)
C7—N1—S1	125.13 (16)	C9—C10—N2	117.5 (2)
C7—N1—H1N1	118.7 (16)	C4—C5—C6	120.2 (2)
S1—N1—H1N1	114.6 (16)	C4—C5—H5	119.9
C10—C9—C8	118.6 (2)	C6—C5—H5	119.9
C10—C9—H9	120.7	C5—C6—C1	119.9 (2)
C8—C9—H9	120.7	C5—C6—H6	120.0
C3—C2—C1	119.8 (2)	C1—C6—H6	120.0
C3—C2—Cl	118.35 (19)	C10—C11—C12	117.4 (2)
C1—C2—Cl	121.81 (17)	C10—C11—H11	121.3
C9—C8—C13	119.6 (2)	C12—C11—H11	121.3
C9—C8—C7	121.66 (19)	C5—C4—C3	120.5 (2)
C13—C8—C7	118.69 (19)	C5—C4—H4	119.7
O5—N2—O4	123.9 (2)	C3—C4—H4	119.7
O5—N2—C10	118.5 (2)	O1—S1—O2	118.58 (11)
O4—N2—C10	117.6 (2)	O1—S1—N1	109.09 (10)
C4—C3—C2	120.2 (2)	O2—S1—N1	103.72 (10)
C4—C3—H3	119.9	O1—S1—C1	108.43 (10)
C2—C3—H3	119.9	O2—S1—C1	108.86 (10)
C13—C12—C11	121.3 (2)	N1—S1—C1	107.65 (11)
C13—C12—H12	119.3		
			
O3—C7—N1—S1	4.0 (3)	O4—N2—C10—C11	3.8 (3)
C8—C7—N1—S1	−175.78 (15)	O5—N2—C10—C9	4.4 (3)
C6—C1—C2—C3	0.4 (3)	O4—N2—C10—C9	−174.4 (2)
S1—C1—C2—C3	−173.82 (19)	C4—C5—C6—C1	−0.1 (4)
C6—C1—C2—Cl	−179.23 (18)	C2—C1—C6—C5	−0.3 (3)
S1—C1—C2—Cl	6.5 (3)	S1—C1—C6—C5	174.2 (2)
C10—C9—C8—C13	0.9 (3)	C9—C10—C11—C12	−1.6 (3)
C10—C9—C8—C7	−178.37 (19)	N2—C10—C11—C12	−179.7 (2)
O3—C7—C8—C9	−149.7 (2)	C13—C12—C11—C10	0.7 (3)
N1—C7—C8—C9	30.1 (3)	C6—C5—C4—C3	0.5 (4)
O3—C7—C8—C13	31.0 (3)	C2—C3—C4—C5	−0.4 (4)
N1—C7—C8—C13	−149.2 (2)	C7—N1—S1—O1	48.2 (2)
C1—C2—C3—C4	0.0 (4)	C7—N1—S1—O2	175.44 (18)
Cl—C2—C3—C4	179.6 (2)	C7—N1—S1—C1	−69.3 (2)
C11—C12—C13—C8	0.9 (4)	C6—C1—S1—O1	5.5 (2)
C9—C8—C13—C12	−1.7 (3)	C2—C1—S1—O1	179.83 (18)
C7—C8—C13—C12	177.6 (2)	C6—C1—S1—O2	−124.79 (18)
C8—C9—C10—C11	0.8 (3)	C2—C1—S1—O2	49.5 (2)
C8—C9—C10—N2	178.95 (19)	C6—C1—S1—N1	123.40 (18)
O5—N2—C10—C11	−177.3 (2)	C2—C1—S1—N1	−62.3 (2)

### Hydrogen-bond geometry (Å, º)

Cg is the centroid of the nitrobenzene ring.

**Table:**

*D*—H···*A*	*D*—H	H···*A*	*D*···*A*	*D*—H···*A*
N1—H1*N*1···O2^i^	0.81 (2)	2.15 (2)	2.954 (3)	169 (2)
C11—H11···O3^ii^	0.93	2.50	3.411 (3)	166
C6—H6···*Cg*^iii^	0.93	2.72	3.550 (3)	150

Symmetry codes: (i) −*x*, −*y*, −*z*+1; (ii) −*x*−1/2, *y*−1/2, *z*; (iii) −*x*+1, *y*−1/2, −*z*+1/2.

## References

[bb1] Bruker (2009). *APEX2*, *SADABS*, *SAINT-Plus* and *XPREP* Bruker AXS Inc., Madison, Wisconsin, USA.

[bb2] Gowda, B. T., Foro, S., Suchetan, P. A. & Fuess, H. (2009). *Acta Cryst.* E**65**, o2516. 10.1107/S1600536809037222 PMC2970249 21577963

[bb3] Gowda, B. T., Foro, S., Suchetan, P. A. & Fuess, H. (2010). *Acta Cryst.* E**66**, o794. 10.1107/S1600536810008731 PMC2984022 21580633

[bb4] Macrae, C. F., Bruno, I. J., Chisholm, J. A., Edgington, P. R., McCabe, P., Pidcock, E., Rodriguez-Monge, L., Taylor, R., van de Streek, J. & Wood, P. A. (2008). *J. Appl. Cryst.* **41**, 466–470.

[bb5] Sheldrick, G. M. (2008). *Acta Cryst.* A**64**, 112–122. 10.1107/S0108767307043930 18156677

[bb6] Suchetan, P. A., Foro, S. & Gowda, B. T. (2011). *Acta Cryst.* E**67**, o930. 10.1107/S1600536811009913 PMC3100054 21754200

[bb7] Suchetan, P. A., Foro, S. & Gowda, B. T. (2012). *Acta Cryst.* E**68**, o1507. 10.1107/S1600536812016765 PMC3344615 22590377

